# Social determinants of smoking among school adolescents in Beijing, China

**DOI:** 10.18332/tid/152202

**Published:** 2022-08-31

**Authors:** Xi Cheng, Xin Guo, Chenggang Jin

**Affiliations:** 1 School of Social Development and Public Policy, Beijing Normal University, Beijing, People’s Republic of China; 2 Chinese Center for Disease Control and Prevention, Beijing, People's Republic of China; 3 Research Center for Health and Social Policy, Beijing Normal University, Zhuhai, People’s Republic of China

**Keywords:** smoking, adolescents, determinants, teachers, peers

## Abstract

**INTRODUCTION:**

Understanding the social determinants that influence adolescent smoking behavior has a meaningful impact on adolescent health. Few studies have simultaneously analyzed the impact of teacher smoking and peer smoking on adolescent smoking. Therefore, the present study aims to investigate the impact of teacher smoking, peer smoking, and other social factors, on adolescent smoking.

**METHODS:**

The participants were drawn from schools in Beijing, China, in 2011, 2013, and 2015, using a multi-stage random group sampling method. The number of schools selected for each year was 160. The study participants were 57240 adolescents aged 8–19 years. The generalized linear model with a binomial distribution and logarithmic link function was used to estimate the influence of social determinants on adolescent smoking behavior.

**RESULTS:**

The results show that both teacher smoking and peer smoking were significantly associated with adolescent smoking. Compared to adolescents whose teachers did not smoke, the prevalence ratio (PR) was 1.28 for adolescents with one teacher who smoked and 1.34 for adolescents with two or more teachers who smoked (95% CI: 1.16–1.41/1.23–1.46, p<0.001/0.001, respectively). Compared to adolescents whose peers did not smoke, the prevalence ratio (PR) was 3.73 for adolescents with one peer who smoked and 8.52 for adolescents with two or more peers who smoked (95% CI: 3.20–4.35/7.48–9.69, p<0.001/0.001, respectively).

**CONCLUSIONS:**

Teacher smoking and peer smoking are significant social determinants of adolescent smoking. Prevention programs should concurrently target peer groups, school settings, and individual students.

## INTRODUCTION

The tobacco epidemic is the leading but preventable cause of morbidity and mortality worldwide. According to the World Health Organization (WHO), approximately 8 million people worldwide die each year as a result of smoking^1^. China is the world’s largest producer and consumer of tobacco, accounting for more than 44% of the world’s total cigarette consumption^2^. According to the 2018 Global Adult Tobacco Survey (GATS), there are currently more than 300 million smokers in China; approximately 26.6% of Chinese people aged ≥15 years are smokers, and more than half of the men in this age group smoke cigarettes^3^. More than 1 million people die each year in China as a result of smoking-related diseases, and this number will double by 2030 if China does not take significant steps to reverse the present smoking trend^4^.

The younger age group of smokers in China is of particular concern, with adolescent smoking rates rising year after year. Smoking initiation during adolescence increases the likelihood of being a lifelong smoker. At least 60% of smokers started smoking before the age of 18 years^5^. Smoking prevalence among adolescents aged 15–24 years increased from 8.3% in 2003 to 12.5% in 2013, according to two statewide prospective studies in China^4^.

The adolescent period begins at the age of ten (adolescent) and lasts into early adulthood (young adult). This is a critical developmental stage during which the physical, psychological, and social identities of adolescents are undergoing tremendous changes^6^. Adolescents are more prone to become addicted to nicotine because their brains are still developing during adolescence and early adulthood, and quitting after becoming dependent is more difficult^7,8^. Therefore, it is necessary to continuously pay more attention to adolescent smoking.

According to social learning theory^9^, the mere perception of the smoking behavior of role models in the social environment can promote adolescents to mirror these behaviors. During adolescence, two key groups in the socialization environment are teachers and peers^10-12^.

Adolescents spend the majority of their time in schools, and teachers have a significant impact on their development and health practices as they get older. Previous studies suggested that teacher smoking was positively associated with adolescent smoking^10,13,14^. However, no consistent findings have been produced. For example, a recent study in South Korea indicated that the risk of smoking among adolescents would increase if their teachers smoked^15^, whereas other researchers found that higher perceptions of teacher smoking were solely associated with student smoking among girls^16^. Consequently, how adolescent smoking behavior is influenced by teachers remains to be elucidated.

The impact of peer smoking on adolescent smoking behavior has been widely documented^17^. For example, a longitudinal study found that peer effects are important determinants of adolescent smoking behavior even after controlling for potential biases in the data^18^. According to a study conducted in 10 nations, peer smoking increases the probability of adolescent smoking by 3% to 6.9%^11^.

Age, sex, class level, pocket money, parental smoking status, etc. are frequently used as indicators of determinants of smoking in adolescents^19^. While some studies have shown that teacher smoking is a determinant of adolescent smoking, and others have shown that peer smoking is a determinant of adolescent smoking, few studies have examined both at the same time^10,15,20^. Our model included both teacher smoking and peer smoking simultaneously to avoid omitted variable bias.

Therefore, the objective of the present study was to investigate social determinants of smoking among school adolescents, with a focus on teacher smoking and peer smoking.

## METHODS

### Sample

The study design was a school-based cross-sectional survey. The participants were drawn from schools in Beijing, China, in 2011, 2013, and 2015. In this study, samples from these three years were pooled. The probability proportional to size (PPS) method was applied where the sampling unit was the school. Schools in the present study were classified into four types: primary schools, junior high schools, ordinary high schools, and vocational high schools. Multi-stage cluster samplings were used to select 2–5 schools from 8 districts in Beijing. Simple random sampling was employed to select 4–7 classes in each school. The number of districts in 2011, 2013 and 2015 was 18, 18, and 16, respectively; and the number of schools in 2011, 2013 and 2015, were 160. Inclusion criteria for the current study included: 1) in the selected classes; 2) attending 4th to 12th grades; 3) attending school on the day of the survey; and 4) willing to participate in this study. A total of 57240 students were recruited in the present study.

### Procedures

Based on informed consent, a class-based, anonymous survey method was adopted. The survey was self-administered in the presence of a teacher and the questionnaire was tested beforehand. None of the adolescents refused to complete the questionnaire. Non-response only occurred in cases when the adolescent did not attend school on the day of the survey. The questionnaire collected information on sociodemographic factors (sex, age, class level, and daily pocket money), adolescent smoking-related behaviors (adolescent current smoking), teacher smoking, peer smoking, and parental smoking. Data collectors were trained to give participants uniform and clear instructions. All data were entered into Epidata 3.1 twice, and cross-validation was conducted to ensure accuracy.

### Measures

Table 1 lists the definitions of the dependent variables and independent variables.

**Table 1:** Definition of explanatory variables used in the analysis

*Variables*	*Definition and coding*
**Dependent variables**	
Current smoking	1 = Having smoked at least a complete cigarette in the past 30 days
	0 = otherwise
**Independent variables of interest**	
Teacher smoking	1 = No teachers smoke
	2 = Only one teacher smokes
	3 = Two or more teachers smoke
Peer smoking	1 = No peers smoke
	2 = Only one peer smokes
	3 = Two or more peers smoke
**Control variables**	
Year	1 = 2011
	2 = 2013
	3 = 2015
Sex	1 = Male
	2 = Female
Class level	1 = Elementary school
	2 = Middle school
	3 = High school
	4 = Vocational high school
Daily pocket money (RMB)	1= <3
	2 = 3– 10
	3 = >10
Parental smoking status	1 = Only father smokes
	2 = Only mother smokes
	3 = Both parents smoke
	4 = Neither parent smokes

RMB: 100 Chinese Renminbi about 15 US$.

*Dependent variables*

Current smoking. Based on the definition of smoking standards recommended by the WHO^21^, respondents indicating that they had smoked at least a complete cigarette in the past 30 days were defined as current smokers.

*Main determinants*

Teacher smoking. Respondents were asked to indicate the number of smoking teachers. Response options ranged from 0= ‘zero’ to 5= ‘five or more’. Then, the responses were divided into three categories: 1) no teachers smoke; 2) one teacher smokes; and 3) two or more teachers smoke.

Peer smoking. Respondents were asked to indicate the number of their five closest friends or classmates who had smoked in the last 30 days, with response options ranging from 0–5. Then, the responses were divided into three categories: 1) no peers smoke; 2) one peer smokes; and 3) two or more peers smoke.

### Control variables

Based on an extensive literature review and availability in the dataset, control variables included: gender (female/male), class level (elementary school, middle school, high school, vocational high school), daily pocket money (<3; 3–10; >3 RMB; with 100 Chinese Renminbi about 15 US$), and parental smoking (only father smokes, only mother smokes, both parents smoke, neither parent smokes). Besides, the year was included in the control variables and was set as a dummy variable.

### Statistical analysis

The database was established using Epidata 3.1. Analyses were performed using Stata (version 17.0; StataCorp, College Station, Texas, USA). Datasets from three surveys (2011, 2013, 2015) were pooled with a *year* dummy variable. List-wise deletion was performed to achieve a sample with complete data. The generalized linear model with a binomial distribution and logarithmic link function was carried out to estimate the association between adolescent smoking status and each independent variable. The reason to use the log-binomial regression model is that it can estimate the prevalence ratio (PR) directly, rather than the odds ratio (OR), which is difficult to interpret precisely^22,23^. A p<0.05 was used as the level of statistical significance.

## RESULTS

### Sample characteristics

Table 2 presents the characteristics of the sample population. The sample had a mean age of 15 years (range: 8–19) and was evenly split by gender. Of the total participants, 5.83% reported that they were current smokers, 40.58% reported that their teachers smoked, and 15.92% reported that their peers smoked. The participants comprised elementary students (31.69%), middle school students (29.12%), high school students (25.39%), and vocational high school students (13.80%); their smoking prevalence was 1.44%, 2.60%, 5.67%, and 23.09%, respectively. Approximately 60% of the participants had 3 RMB or more pocket money each day, and over half had parents who smoked.

**Table 2:** Characteristics of the study sample (2011–2015)

*Characteristics*	*n*	*%*
**Total**	57240	100
*Dependent variables*		
**Current smoking**		
Yes	3339	5.83
No	53901	94.17
*Independent variables of interest*		
**Teacher smoking**		
No teachers smoke	33865	59.42
Only one teacher smokes	11276	19.78
Two or more teachers smoke	11855	20.80
**Peer smoking**		
No peers smoke	38406	84.08
Only one peer smokes	2584	5.66
Two or more peers smoke	4690	10.27
*Control variables*		
**Year**		
2011	16504	28.83
2013	18322	32.01
2015	22414	39.16
**Sex**		
Male	28676	50.18
Female	28473	49.82
**Class level**		
Elementary school	18114	31.69
Middle school	16644	29.12
High school	14512	25.39
Vocational high school	7890	13.80
**Daily pocket money** (RMB)		
<3	17517	30.60
3–10	18651	32.58
>10	21072	36.81
**Parental smoking status**		
Only father smokes	30352	53.07
Only mother smokes	533	0.93
Both parents smoke	1866	3.26
Neither parent smokes	24443	42.74

RMB: 100 Chinese Renminbi about 15 US$.

### The effects of teacher smoking and peer smoking on adolescent current smoking

**Figure 1:**
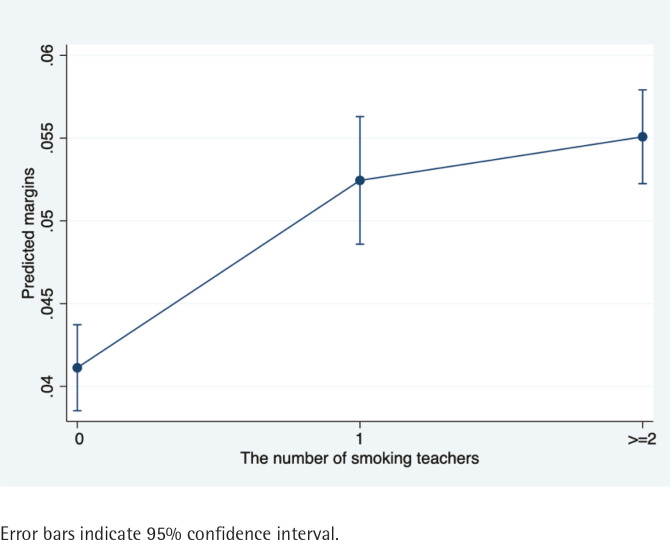
Marginal effects of teacher smoking on adolescent current smoking

Table 3 shows the results of the log-binomial model examining the effects of teacher and peer smoking on current smoking among adolescents. Adolescents with one smoking teacher had a prevalence probability of current smoking that was 0.28 times greater than adolescents whose teachers did not smoke after adjusting for other covariates (PR=1.28; 95% CI: 1.16–1.41, p<0.001). The PR for adolescents with two or more teachers who smoked was slightly higher at 1.34 (95% CI: 1.23–1.46, p<0.001). Figure 1 presents a visual display of the marginal effects of teacher smoking on current smoking among adolescents. The results suggest that the probability of adolescents becoming current smokers would increase slightly as the number of smokers among their teachers increased.

**Table 3:** The influence of teacher smoking and peer smoking on adolescent smoking: The generalized linear model (N=45368)

*Characteristics*	*Current smoking PR (95 % CI )*
**Number of smokers among 5 close classmates or friends**	
0 (Ref.)	1
1	3.730 (3.20–4.35)***
≥2	8.515 (7.48–9.69)***
**Number of teachers who smoke**	
0 (Ref.)	1
1	1.275 (1.16–1.41)***
≥2	1.339 (1.23–1.46)***
**Year of study**	
2011 (Ref.)	1
2013	1.817 (1.31–2.52)***
2015	1.669 (1.20–2.31)**
**Class level**	
Elementary school (Ref.)	1
Middle school	1.059 (0.89–1.26)
High school	1.347 (1.14–1.56)**
Vocational high school	2.792 (2.36–3.30)***
**Sex**	
Female (Ref.)	1
Male	2.305 (2.08–2.55)***
**Daily pocket money** (RMB)	
<3 (Ref.)	1
3–10	1.076 (0.93–1.23)
>10	1.307 (1.15–1.49)***
**Parents who smoke**	
Neither parent smokes (Ref.)	1
Only father smokes	1.369 (1.26–1.49)***
Only mother smokes	1.437 (1.17–1.77)**
Both parents smoke	1.570 (1.40–1.77)***

PR: prevalence ratio. RMB: 100 Chinese Renminbi about 15 US$.

* p<0.05.

** p<0.01.

*** p<0.001.

**Figure 2:**
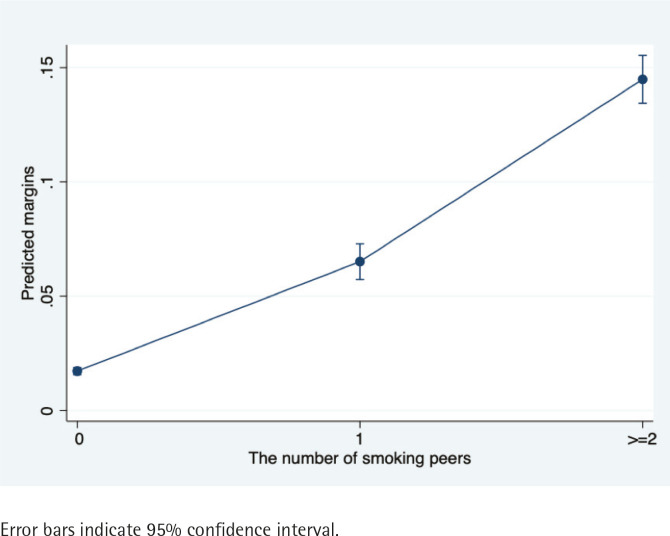
Marginal effects of peer smoking on adolescent current smoking

Adolescents with one close classmate or friend who smoked had a prevalence of current smoking that was 2.73 times greater than adolescents with no close classmates or friends who smoked after adjusting for other covariates (PR=3.73; 95% CI: 3.20–4.35, p<0.001). Adolescents with two or more smoking close classmates or friends had a prevalence of current smoking that was 7.52 times greater than adolescents whose close classmates or friends did not smoke after adjusting for other covariates (PR=8.52; 95% CI: 7.48–9.69, p<0.001). Figure 2 gives a visual display of the marginal effects of peers’ smoking behaviors on current smoking among adolescents. The results indicate that as the number of peers who smoke increases, the probability of adolescents becoming current smokers increases.

### The effects of other determinants on adolescent current smoking

As shown in Table 3, the prevalence of being current smokers of adolescents in 2015 was 0.82 times greater than that in year 2011 after adjusting for other covariates (PR=1.82; 95% CI: 1.31–2.52, p<0.001). Adolescents from high schools/vocational high schools had a prevalence of being current smokers that was 0.35/1.79 times greater than adolescents from elementary schools (PR=1.35/2.79; 95% CI: 1.14–1.56/2.36–3.30, p<0.01/0.001, respectively). Boys had a prevalence of being current smokers that was 1.31 times greater than girls (PR=2.31; 95% CI: 2.08–2.55, p<0.001). Parental smoking was statistically significantly associated with current smoking among adolescents; adolescents whose father only smoked had a PR of 1.37 (95% CI: 1.26–1.49, p<0.001), those whose mother only smoked had a PR of 1.44 (95% CI: 1.17–1.77, p<0.01), and those whose parents both smoked had a PR of 1.57 (95% CI: 1.40–1.77, p<0.001). Adolescents who had more than 10 RMB as daily pocket money had a prevalence of being current smokers that was 0.31 times greater than those who had 3 RMB or less (95% CI: 1.15–1.49, p<0.001).

## DISCUSSION

This study found that teacher smoking, peer smoking, parental smoking, gender, class level, and daily pocket money were important social determinants of adolescent smoking behavior. Adolescents had a greater prevalence ratio of being current smokers as the number of smokers among their teachers, peers and parents increased.

Our findings are in line with the mainstream research results. A number of studies reported a positive association between teacher smoking and adolescent smoking^24,25^. For example, a study conducted in Spain found that the higher the levels of visibility of teacher smoking, the more likely a middle school or high school student is to be a smoker^10^. Teacher smoking was also found to be the determinant of smoking among elementary school students^26^. However, no consistent findings have been produced. For instance, a study in Taiwan found that the effect of smoking teachers on adolescents’ smoking behavior was increased only when friends did not smoke. When friends’ smoking status was included in the model, the relationship between teacher smoking and adolescent smoking was reversed^27^. The disparities in empirical results may be due to differences in the measurement of key variables, measurement methods, study subjects, and cultural context. Our findings, which suggest that the increase in the number of teachers who smoke would increase the prevalence ratio of being a smoker in adolescents, help to understand the social influences from teachers on adolescent smoking.

The observed associations between teacher smoking and adolescent smoking can be attributed to social cognitive theory^28^. According to social cognitive theory, the mere observation of role models performing a behavior can promote observers to engage in the behavior they already learned. As influential role models, if teachers themselves smoke, they would make students more likely to perceive smoking as something positive and acceptable. In other words, the perceived smoking behaviors of teachers would make it easier for students to smoke or attempt to smoke. Another likely explanation is that a supportive environment will enable behavioral changes, according to the Ottawa Charter for Health Promotion^29^. Teacher smoking is a proxy variable for the smoking environment in the school setting. In this case, teacher smoking serves as enabling support to promote adolescent smoking. Thus, it is important for schools to prohibit teachers from smoking in school areas.

In this study, peer smoking was found to be significantly associated with adolescent smoking. This finding is consistent with many other studies on the association between peer smoking and smoking among adolescents at different school grade levels^18,30,31^. Although estimates of peer impacts on adolescent smoking differ by country^18,30^, all concluded that peer smoking is an important determinant of adolescent smoking.

The reasons for the observed influence of peers’ smoking behaviors on adolescent smoking can be attributed to the following aspects. First, social cognitive theory^32^ emphasizes the importance of cognitive representations in the form of expectations about social norms that arise from observational and experiential learning. Adolescents with peers who smoke have easier access to cigarettes and consider smoking as unrestricted and normative. Second, according to social identity theory^33^, if their peer groups favor smoking, then adolescents will smoke to remain in good standing or gain a sense of belonging. Therefore, health promotors in schools should develop correct social norms regarding adolescent smoking behaviors and tobacco control policies for peer groups in addition to tobacco control policies for individual adolescents.

The positive association between parental smoking and adolescent smoking was well documented^34-36^. The present study had results similar to the previous studies where parental smoking was reported as a strong predictor of adolescent smoking. The key role of parents as important socialization figures for adolescent smoking can also be explained by Social Cognitive Theory^28^.

In this study, gender, class level, and daily pocket money were also reported as social determinants of smoking. These findings are consistent with many other studies on the association of gender^37^, class level^38^, and daily pocket money^39^, and adolescent smoking. Having more disposable income can serve as an attribute in the social environment that makes smoking behavior easier to perform^28^.

### Strengths and limitations

This study has several strengths. First, the study used data from three surveys conducted between 2011 and 2015, which were large in size and covered a wide population. Second, through continuous improvement of the monitoring program and system, the survey data were true and reliable and better reflected the tobacco use of primary and secondary school students in Beijing. Third, the log-binomial regression model we used can directly estimate PR, which is easier to understand than OR. However, the present study has several limitations. First, we relied on self-report measures, which may be subject to recall bias and social desirability effects. Second, although our sample size was considerably large, the study was cross-sectional in design, thus making it hard to infer causality like longitudinal data. Third, because districts and schools kept changing in Beijing from 2011 to 2015, we did not conduct multilevel analysis. Therefore, we failed to control heterogeneity between clusters.

## CONCLUSIONS

Teachers’ and peers’ smoking behaviors were found to be significantly associated with adolescent smoking behavior. In China, many current antismoking programs targeting adolescents only provide individual students with information about the harmful effects of smoking. Such interventions that attempt to change health beliefs showed small and short-term effects. Multilevel interventions that target school settings, family settings, peer groups, and individuals may work better to change behaviors. Besides, differences in the determinants of smoking across school grade levels should also be considered when designing and implementing antismoking programs in schools.

## Data Availability

The data supporting this research cannot be made available for privacy or other reasons.
